# A pilot study on spatial hearing in children with congenital unilateral aural atresia

**DOI:** 10.3389/fped.2023.1194966

**Published:** 2023-08-09

**Authors:** Hanna Josefsson Dahlgren, Cecilia Engmér Berglin, Malou Hultcrantz, Filip Asp

**Affiliations:** ^1^ Department of Clinical Science, Intervention and Technology, Karolinska Institutet, Stockholm, Sweden; ^2^ Department of Otorhinolaryngology, Karolinska University Hospital, Stockholm, Sweden

**Keywords:** unilateral aural atresia, unilateral conductive hearing loss, UCHL, sound localization, speech recognition, BCD, bone conduction device, early fitting

## Abstract

Despite normal hearing in one ear, individuals with congenital unilateral aural atresia may perceive difficulties in everyday listening conditions typically containing multiple sound sources. While previous work shows that intervention with bone conduction devices may aid spatial hearing for some children, testing conditions are often arranged to maximize any benefit and are not very similar to daily life. The benefit from amplification on spatial tasks has been found to vary between individuals, for reasons not entirely clear. This study has sought to expand on the limited knowledge on how children with unilateral aural atresia recognize speech masked by competing speech, and how horizontal sound localization accuracy is affected by the degree of unilateral hearing loss and by amplification using unilateral bone conduction devices when fitted before 3 years of age. In a within-subject, repeated measures design, including 11 children (mean age = 7.9 years), bone conduction hearing device (BCD) amplification did not negatively affect horizontal sound localization accuracy. The effect on speech recognition scores showed greater inter-individual variability. No benefit from amplification on a group level was found. There was no association between age at fitting and the benefit of the BCD. For children with poor unaided sound localization accuracy, there was a greater BCD benefit. Unaided localization accuracy increased as a function of decreasing hearing thresholds in the atretic ear. While it is possible that low sound levels in the atretic ear provided access to interaural localization cues for the children with the lowest hearing thresholds, the association has to be further investigated in a larger sample of children.

## 1. Introduction

Individuals with unilateral conductive hearing loss due to unilateral aural atresia (UAA) report a high degree of difficulties in tasks related to binaural hearing, such as sound localization and recognition of speech in noise (1). Treatment using bone conduction hearing devices (BCDs) aim to restore hearing in the atretic ear and aid in binaural hearing. From infancy, children might be offered a passive transcutaneous BCD fitted on a softband. The standard percutaneous skin-penetrating BCD attached to a titanium screw osseointegrated in the cortical bone superior and posterior to the pinna provide the user with higher amplification compared to the softband (2). It has been the first hand choice for treating hearing loss in UAA at our clinic for several years as surgery is minimally invasive and serious adverse events are rare. However, in a study from 2015, authors found that 47% of children implanted with a percutaneous BCD had discontinued using the implant 5 years after surgery (3). Insufficient benefit from amplification was one of the most commonly stated reasons for non-usage (3). Pure-tone thresholds improve from amplification (4, 5) but the effect on binaural hearing needs to be investigated further. There seem to be a large inter-individual variability on the effect from amplification on speech recognition thresholds (SRTs) (6, 7) as well as on sound localization accuracy (SLA) (6, 8). It is known that some individuals localize fairly good in the monaural unaided setting and thus will not benefit as much from BCD (6, 8, 9). A possible explanation for the high inter-individual variability in benefit from a BCD is that some individuals with congenital UAA learn to use monaural spectral cues for localization in the horizontal plane (10). The age at which the child is fitted with the BCD has also been suggested as a factor influencing the benefit from amplification (11, 12). In children with unilateral sensorineural hearing loss, sound localization accuracy seems to improve for children fitted with a hearing aid by 5 years of age, whereas not for children fitted by 9 years of age (13). In most studies regarding children implanted with a BCD, the study participants have started using their BCD at 4–6 years of age at the earliest (9). Studies presenting results from surgically implanted percutaneous or transcutaneous devices rarely disclose whether the participants in the studies have previously been using a different system for bone conduction and for how long. As binaural hearing and the central auditory pathways develop during the first 5–6 years of life (14–16), early treatment might be beneficial on binaural tasks such as horizontal sound localization and speech recognition in acoustically challenging conditions (11, 12).

## 2. Aim

The aim of the present study was to quantify the effect of early access to unilateral bone conduction amplification on sound localization accuracy and recognition of speech in symmetrically separate competing speech in a cohort of children with UAA fitted with a BCD before 3 years of age.

## 3. Materials and methods

### 3.1. Study participants

**Figure 1:**
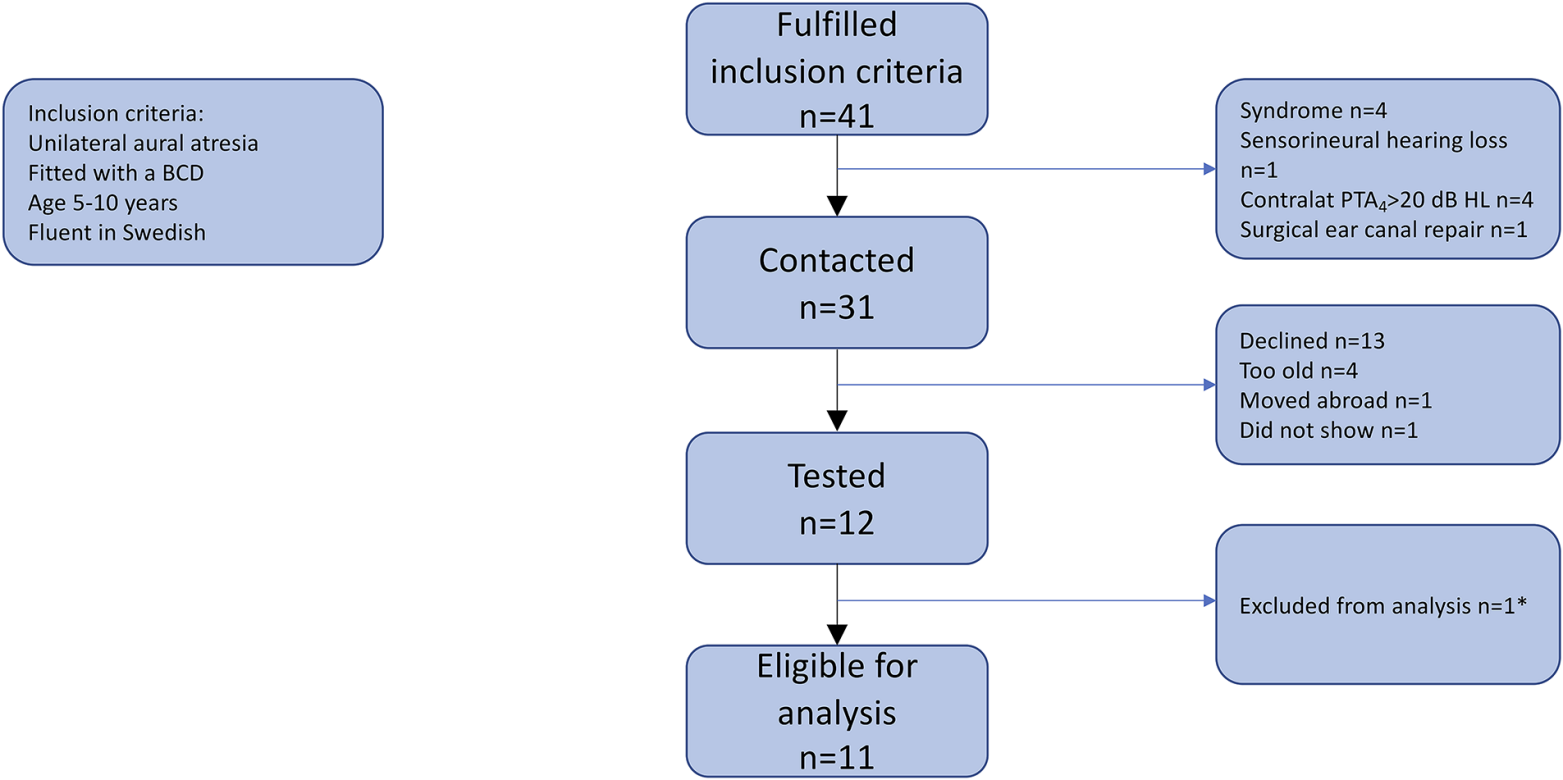
Flow chart for inclusion in the study. Thirteen eligible participants declined participation due to different reasons. Four patients were between 5 and 10 years old in 2018 but could not make it to any of the appointments available at that time and had grown too old to include in the study when more opportunities opened in 2021. *One participant did not perform any aided measurements due to lack of time and was therefore excluded from analysis.

Children with congenital UAA were recruited from a list of patients that had attended the atresia clinic at the Hearing Habilitation Unit at Rosenlund’s Hospital from 2015 to 2017. Forty-one individuals were eligible based on the following inclusion criteria: 5–10 years of age, unilateral congenital atresia, fitted with a BCD, and fluent in the Swedish language. Ten subjects were excluded based on the following exclusion criteria: syndrome-associated atresia (*n* = 4); sensorineural hearing loss (*n* = 1); contralateral air conduction pure-tone average across 500, 1,000, 2,000, and 4,000 Hz (PTA_4_) >20 dB hearing level (HL) (*n* = 4); and surgical ear canal repair (*n* = 1). Investigations took place during January 2018, November 2020, and spring 2021. Four individuals that were not able to come in for testing in 2018 had grown too old for inclusion in 2020. One subject had moved abroad and could not be contacted. Thirteen individuals declined or could not attend any of the visits for different reasons. One subject repeatedly did not show up for measurements. One subject did not fulfill any aided measurements due to lack of time and was excluded from analysis (Figure 1).

### 3.2. Study design

In a 3 h visit, recognition of speech in spatially separate competing speech and horizontal sound localization accuracy were tested in a within-subject repeated measures design (aided and unaided, test order was pseudo-randomized based on the last digit of the subject's national ID number). Aided and unaided pure-tone hearing thresholds were also measured. The children used their own BCD, either a Cochlear or an Oticon processor (Table 1), for all aided measurements. The devices had been previously programmed using the fitting software provided by the manufacturer and had been fine-tuned according to the preferences of the child. Background data were retrieved from the caregiver of the study participants and from patient charts. Data on mean usage per day were retrieved from the device using the fitting software. The study participants had been provided with a copy of Parents Evaluation of Aural/Oral Performance of Children (PEACH) to fill out before the visit. Ethical approval was obtained from the regional ethics committee in Stockholm, 2012/1661-31/3. Written consent was acquired from all study participants.

**Table 1:** Background data including type of device, age, gender, and degree of usage from the computer log of the BCD.

Subject ID	Device	Processor	Age at fitting (year)	Age at testing (year)	Duration of device use (year)	Usage (h/day)	Gender	Atretic side
1	PC	Baha 4	1.75	7.1	5.7	7.5	M	R
2	PC	Baha 5	2.33	10.1	7.8	5.2	M	R
3	PC	Ponto Pro	2.83	8.1	5.3	—^a^	M	R
4	PC	Baha 5	2.83	8.5	4.8	7.5	M	R
5	Softband	Ponto Pro	0.5	5.3	4.9	6.6	F	L
6	PC	Baha 5	0.42	5.5	5.2	5.7	M	R
7	Softband	Baha 5	1.25	8.5	7.2	5.3	M	R
8	BAHA Attract	Baha 5	0.17	9.9	7.2	0.3	M	R
9	Softband	Baha 5 SP	0.25	5.11	5.6	1.1	F	R
10	PC	Baha 5	2.0	7.8	5.5	4.2	M	R
11	PC	Baha 5	2.33	10.10	8.45	—^a^	M	R
Mean ± SD			1.5 ± 1.0	7.9 ± 1.9	6.3 ± 1.3	4.8 ± 2.6		

PC, percutaneous fixture.

All study participants were initially fitted with a BCD on softband. The time of surgery for a percutaneous device or BAHA attract was unavailable to the authors.

^a^ System for reading the computer log of the device not functioning.

### 3.3. Unaided and aided hearing thresholds

Unaided air- and bone conduction hearing thresholds were measured according to ISO 8253-1 (2010) using TDH39 supra-aural headphones and the Radioear B71 bone transducer. Masking of the non-test ear was applied as appropriate. To estimate the degree of amplification provided by the BCD, aided hearing thresholds were quantified by measuring frequency-modulated tone thresholds in sound field using a fixed-frequency Békèsy technique. While the reliability of this technique is not quantified in children, it is characterized by high reliability and reproducibility in adults (17, 18). During the measurements, the contralateral normal ear was plugged by an earplug (EAR Classic foam earplug; 3M, Minneapolis, MN, USA) and a circum-aural hearing protector (Bilsom 847 NST II, Honeywell Safety Products, RI, USA) was placed over the ear plug. The combination of the plug and the circum-aural hearing protector was previously estimated to provide an average of 39 dB attenuation of the PTA_4_, based on recordings in adults (*n* = 8) with normal hearing according to ISO 4869-1 (1990) (19, 20). Mean (SD) attenuation values as recorded in those adults (*n* = 8) were 34.7 dB (6.3 dB) at 0.5 kHz, 35.1 dB (6.0 dB) at 1 kHz, 40.5 dB (2.9 dB) at 2 kHz, 47.2 dB (5.6 dB) at 3 kHz, 49.4 dB (4.4 dB) at 4 kHz, and 46.2 dB (6.4 dB) at 6 kHz.

### 3.4. Horizontal sound localization ability

An eye-tracking technique was used to determine the perceived sound location. The setup, stimuli, and quantification of sound localization responses have been previously described in detail (21). The rationale for using this test was that it allows for rapid determination of horizontal localization accuracy (approximately 3 min recording time) and has previously been used for measuring sound localization accuracy in children with unilateral hearing loss (22) as well as in measuring the difference in performance between bilateral and unilateral sound stimulation (23).

#### 3.4.1. Setup

**Figure 2:**
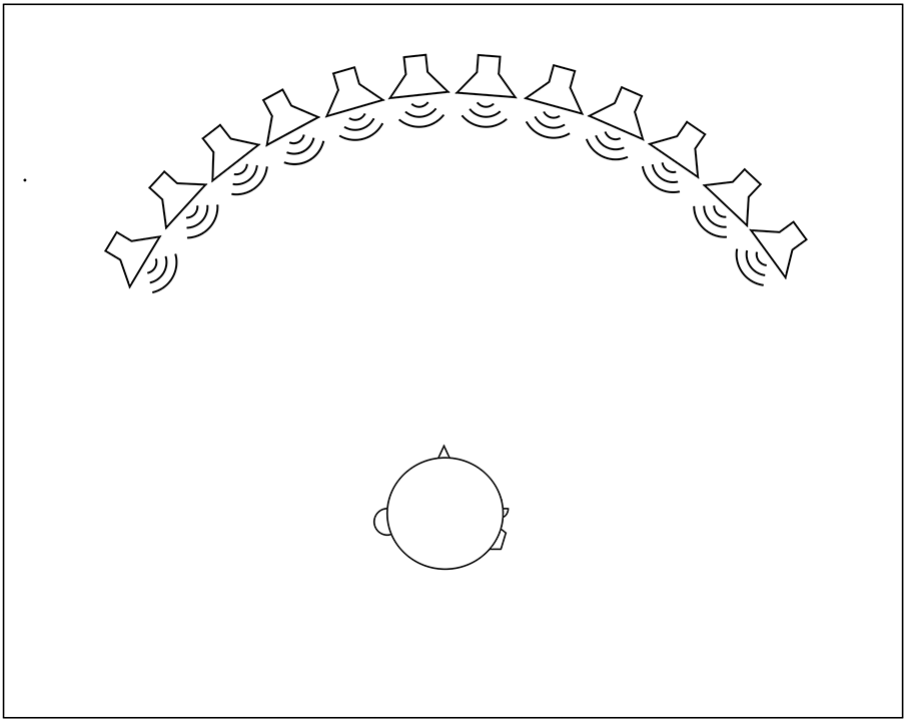
Setup for testing of SLA. Twelve loudspeakers each coupled to a 7-inch video display (LD pairs) were placed in a 110° arc in the frontal horizontal plane relative to the subject who was seated facing the loudspeaker array. The distance from the LD pairs to the head of the study participant was approximately 1.2 (loudspeaker) and 1.1 m (screen). The LD pairs were adjusted to be at ear level of the study participant.

Measurements were conducted in a double-walled sound booth [ambient sound level = 25 dBA, reverberation time T_30_ = 0.11 s at 500 Hz, as recorded with a B & K 2238 Mediator and a B & K 2260 Investigator (Brüel & Kjær), respectively]. Twelve active loudspeakers each coupled to a 7-inch video display (LD pairs) were placed equidistantly in a 110° arc in the frontal horizontal plane, resulting in loudspeaker positions at ±55°, ±45°, ±35°, ±25°, ±15°, and ±5° relative to the subject who was seated facing the loudspeaker array. The distance from the LD pairs to the head of the study participant was approximately 1.2 (loudspeaker) and 1.1 m (screen). The LD pairs were vertically adjusted to the height of the study participant using a motorized stand, situating the loudspeakers at ear level (Figure 2).

To record the gaze of the study participants in relation to the LD pairs, an eye-tracking system was used (Smart Eye Pro, Smart Eye AB, Gothenburg, Sweden). The coordinates of the LD pairs were defined in three dimensions in the eye-tracking system, resulting in areas of interest [AOIs (21, 24)]. Twelve AOIs (width = 0.17 m; height 0.55 m) constituted a continuous array of AOIs in a 3D model, corresponding to the physical LD pairs.

#### 3.4.2. Stimulus

The visual stimulus was a colorful children's cartoon. The auditory part of the stimulus consisted of a broadband musical melody with a long-term frequency spectrum similar to that of a female voice and naturally occurring amplitude modulations. The stimulus was presented at 63 dB sound pressure level (SPL) (A). The rationale for using this stimulus was that it allows comparison with previous findings in children and adults with normal hearing (21), with children with congenital unilateral sensorineural hearing losses (22), and with adults with congenital unilateral atresia (25).

#### 3.4.3. Test procedure and quantification of localization responses

Study participants were familiarized with the auditory–visual stimulus during a gaze-calibration procedure in which the stimulus was presented from different azimuths. The test started by presenting the stimulus from the LD pair at −5°. After approximately 7 s, the visual stimulus was stopped, and the sound immediately shifted to a randomized loudspeaker. After 1.6 s, the visual stimulus was reintroduced at the azimuth of the sounding loudspeaker. Azimuthal shifts were repeated 24 times following a beforehand generated order of randomized shifts.

Children were allowed to move their head freely. They were instructed to look where they perceived the sound was coming from and informed that they would be guided by audition only during sound-only presentation and that the visual part of the stimulus would reappear at the same azimuth as the sound.

The position of the study participant's pupil relative to the LD pairs was sampled at 20 Hz during the 1.6 s sound-only periods. The median pupil position from the last 500 ms of the sound-only period was defined as the perceived sound source location. SLA was quantified as an error index (EI) [for calculations, see Asp et al. (21)] ranging from 0 to 1, where 0 represents a perfect performance and 1 a random performance. Based on test–retest analyses in infants and young children, also from Asp et al. (21), a within-subject difference in the EI of ±0.12 was considered statistically significant at the 95% confidence level.

### 3.5. Speech recognition thresholds in competing speech

**Figure 3:**
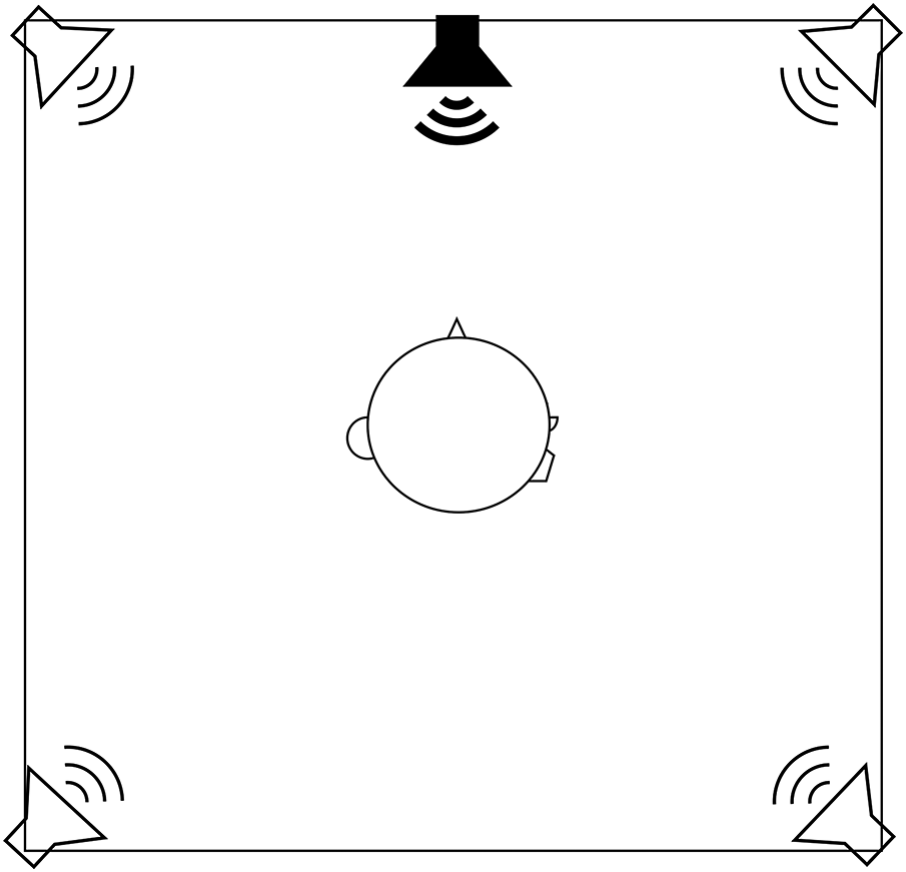
Setup for testing of SRT. Participants were seated facing a loudspeaker presenting target speech at 0° azimuth. Interfering speech was presented from four spatially symmetrically separated loudspeakers at ±30° and ±150° azimuth at a fixed overall level of 63 dB SPL measured at the position of the subject's head.

Measurements of SRT were performed in a setup resembling a challenging everyday listening situation (19, 22, 26) using a matrix test (22, 26). Participants were seated in the middle of a double-walled sound booth facing a loudspeaker presenting target speech at 0° azimuth. Interfering speech was presented from four spatially and symmetrically separated loudspeakers at ±30° and ±150° azimuth at a fixed overall level of 63 dB SPL measured at the position of the subject's head (Figure 3). The interferers comprised four non-correlated recordings of a single male talker reading a novel. The target speech (the Hagerman sentences) was a female voice (27). Each sentence consisted of five words that formed a grammatically correct sentence with low semantic predictability in a fixed syntax (e.g., “Peter höll nio nya lådor,” in translation: “Peter held nine new boxes”).

Study participants were instructed to face the loudspeaker presenting target speech and asked to repeat the sentences from three lists (one training list), each containing 10 sentences. This resulted in the presentation of 30 sentences per listening condition (aided and unaided). No sentence was repeated. Oral responses were recorded and scored by an audiologist outside the test room. Both the aided and unaided assessments started by presenting the first sentence of the training list at a signal-to-noise ratio (SNR) of +10 dB. For the following training sentences, the target speech level decreased up to three times in 5 dB steps, then up to three times in 3 dB steps, and then in 2 dB steps until the number of correct words in a sentence was ≤2. When the number of correct words in a sentence was ≤2, or the training list ended, the training was terminated. Subsequently, two lists (i.e., 20 sentences) were presented. The level adjustment of the target speech aimed at a threshold of 40% words correctly repeated according to the following scheme: the target speech level was changed +2 dB for zero correctly identified words, +1 dB for one correctly identified word, 0 dB for two correctly identified words, −1 dB for three correctly identified words, −2 dB for four correctly identified words, and −3 dB for five correctly identified words. The 40% threshold and the adaptive scheme for level adjustment were based on computer simulations and analysis of the maximum steepness of the psychometric function (27–29). The SRT was defined as the mean of the SNRs for the last 10 of the totally 20 presented sentences (29, 30). The mean (SD) test–retest difference for this task was previously estimated in adults to 1.1 dB (1.4 dB) (26).

### 3.6. Subjective assessment

The PEACH score is a questionnaire consisting of 13 questions assessing auditory behaviors of the child in different situations and is to be filled in by the caregiver. It is divided into two domains, quiet and noise. The questionnaire was developed from the more extensive PEACH diary (31) and is validated in Swedish (32). It is also used in the national pediatric hearing register in Sweden. The questionnaire was sent home to the study participants in advance along with written instructions on how to fill out the form. If the caregiver had not filled out the form at home, the caregiver was allowed to fill out the questionnaire on the day of testing.

### 3.7. Statistical calculations

PTA_4_ were calculated as the mean of hearing thresholds at 500, 1,000, 2,000, and 4,000 Hz. Statistical calculations were conducted using GraphPad Prism 9.3.1 (350). Correlations with age, age at first fitting of the BCD, mean time of usage per day, duration of device use, and unaided PTA_4_ as predictor variables and SLA and SRT as dependent variables were performed using non-parametric tests (Spearman correlation) due to skewed distribution of the data. Paired comparisons (unaided vs. aided listening) were also performed using non-parametric tests (Wilcoxon signed rank).

## 4. Results

The final sample comprised 11 children aged 5.3–10.8 years (mean 7.9 years, SD 1.9). All study participants had a normal eardrum on the non-atretic side. Background data are presented in Table 1. For all participants, age of first fitting was decided as when they first started using a BCD on softband mean (SD) of 1.5 (1.8) years. At the time of testing, three individuals used a conventional BCD on softband, one participant used a passive transcutaneous BAHA Attract, and seven individuals used active percutaneous devices. Historical data on device use were not available to the authors. Data on mean time of usage per day since their last control were retrieved from the fitting software. The study participants used their BCD for a mean (SD) of 4.8 (2.6) h per day (Table 1).

### 4.1. Hearing thresholds

The mean (SD) PTA_4_ of the atretic ear was 64.3 (4.9) dB HL (Table 2). All study participants improved their PTA of the atretic ear when using their BCD, resulting in a mean (SD) PTA_4_ of 24.9 (8.8) dB HL. Six of the study participants (subjects 1, 2, 4, 6, 10, and 11) reached aided hearing thresholds <25 dB HL.

**Table 2:** Individual hearing thresholds of the impaired ear.

Subject ID	AC PTA_4_ imp (dB HL)	BC PTA_4_ imp (dB HL)	AC PTA_4_ aided imp (dB HL)	AC PTA_4_ better ear (dB HL)
1	65	5	18.5	6
2	65	11	16.0	0
3	65	11	29.1	8
4	65	0	17.3	5
5	66	11	42.7	3
6	65	10	17.5	9
7	61	4	32.2	6
8	63	10	31.2	5
9	74	9	30.1	5
10	53	0	18.3	3
11	64	4	19.6	4
Max	74	11	42.7	9
Min	53	0	16.0	0
Mean ± SD	64.2 ± 4.9	6.8 ± 4.4	24.8 ± 8.7	4.9 ± 2.5

Imp, impaired ear; PTA_4_, pure-tone average; AC, air conduction threshold; BC, bone conduction threshold.

Air conduction thresholds of the better ear are also presented.

**Figure 4:**
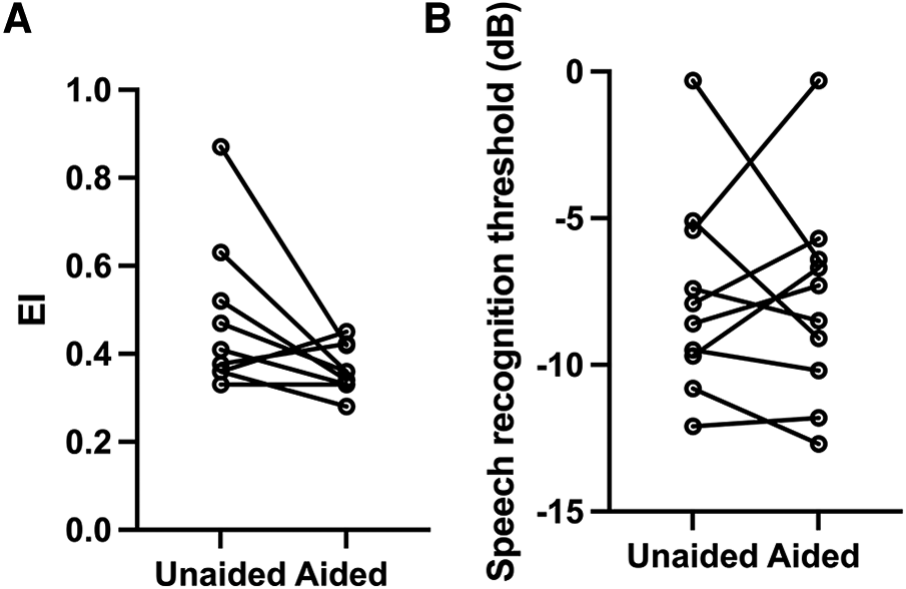
(**A**) Individual values of unaided and aided SLA performance. A lower EI indicates a better performance. There was no significant benefit from amplification on a group level (Spearman correlation, *p* > 0.05). In the aided setting, the three individuals with the highest unaided EI had intra-individual statistically significant benefit compared to the unaided measurements (*p* < 0.05). (**B**) There was a high degree of inter-individual variability on SRT both aided and unaided. A more negative SRT (dB) value indicates a better performance.

Individual results from the sound localization test and speech recognition are summarized in Table 3 and Figure 4.

**Table 3:** Individual results on SLA and SRT, aided and unaided.

Subject ID	AC PTA_4_ (dB HL)^a^	SLA unaided (EI)	SLA aided (EI)	SLA benefit (EI)	SRT unaided (dB)	SRT aided (dB)	SRT benefit (dB)
1	65	0.410	0.330	−0.080	−5.1	−9.1	−4.0
2	65	0.520	0.340	−0.180^b^	−8.6	−7.3	1.3
3	65	—	—	—	−7.4	−8.5	−0.9
4	65	0.470	0.360	−0.110	−9.7	−6.7	3.0
5	66	—	—	—	−5.4	−0.3	4.1
6	65	0.630	0.360	−0.270^b^	—	—	—
7	61	0.330	0.330	0.000	−9.5	−10.2	−2.3
8	63	0.360	0.450	0.090	−10.8	−12.7	−1.9
9	74	0.870	0.420	−0.450^b^	−0.3	−6.4	−5.9
10	53	0.360	0.280	−0.080	−7.9	−5.7	2.2
11	64	0.377	0.423	0.046	−12.1	−11.8	0.3
Max	74	0.87	0.45	−0.450	−12.1	−12.7	−5.9
Min	53	0.33	0.28	0.090	−0.3	−0.3	4.1
Mean ± SD	64.2 ± 4.9	0.48 ± 0.17	0.37 ± 0.05	−0.11 ± 0.17	−7.7 ± 3.4	−7.9 ± 3.5	−0.41 ± −3.18

A more negative SRT value indicates a better performance. There was no significant benefit from amplification on SLA or SRT on a group level (*p* > 0.05, Wilcoxon matched pairs). Three individuals showed significant intra-individual change in SLA when comparing unaided to aided scores (a change of ± 0.12 being statistically significant in infants (*p* < 0.05).

^a^ Unaided air conduction thresholds of the atretic ear.

^b^ Significant intra-individual change *p* < 0.05.

### 4.2. Sound localization accuracy

SLA data from two study participants could not be interpreted and were excluded from further analysis (one participant did not cooperate to testing, possibly due to tiredness; one participant had a congenital eye anomaly that made eye tracking not possible). The mean (SD) of unaided EI was 0.48 ± 0.17, whereas the mean (SD) of aided EI was 0.37 ± 0.05; individual results are plotted in Figure 4A and presented in Table 3. There was no statistically significant difference between the unaided and aided results (*p* = 0.078, *n* = 9, Wilcoxon matched pairs).

Based on previous calculations on test–retest reliability for the localization task, and an estimate of the 95% confidence interval for a single error index value based on this reliability (95% CI = ±0.054 for adults; 95% CI = ±0.12 for infants), we analyzed intra-individual performance differences (unaided vs. aided) in localization accuracy. The three study participants who showed the poorest unaided SLA (participant 2, 6, and 9) showed intra-individual statistically significant improvements when tested with the BCD (±0.12) (*p* < 0.05).

**Figure 5:**
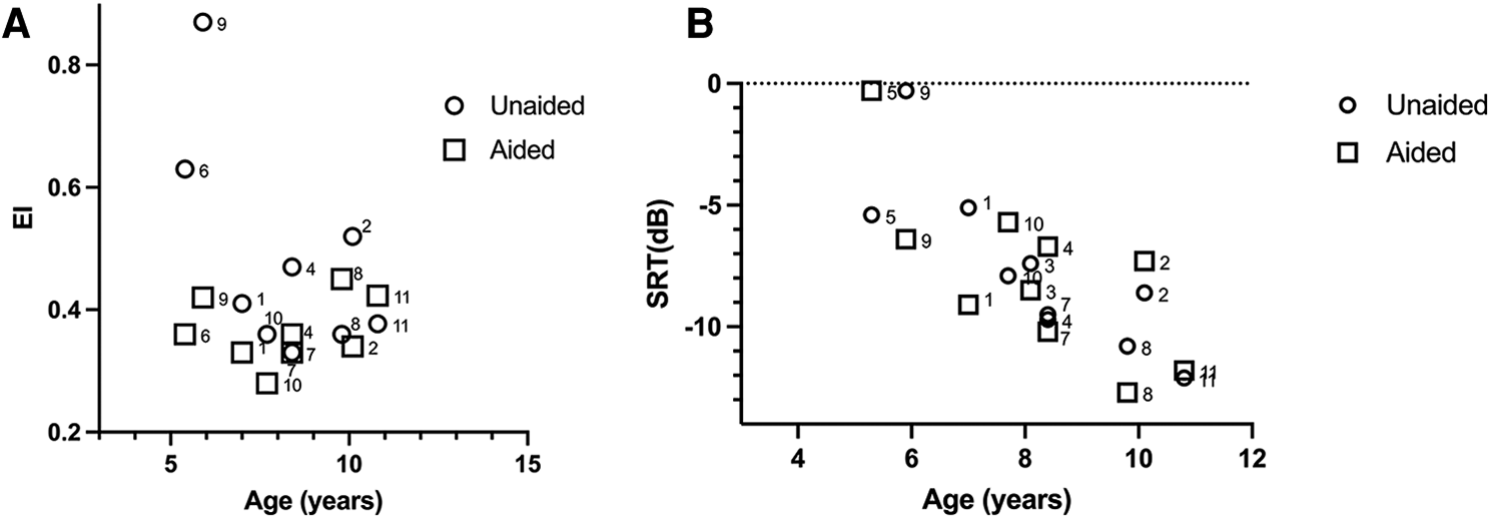
(**A**) SLA performance unaided and aided correlated to age. An EI value of 0 indicates a perfect performance and a value of 1 a random performance. Two of the youngest participants (6 and 9) had the worst unaided SLA performance, but there was no significant correlation between SLA and age. The youngest individuals had the most benefit from amplification. (**B**) SRT correlated with age. A more negative value indicates better speech discrimination. Both aided and unaided SRTs were correlated to age (*p* < 0.05), where the older study participants had lower (better) SRTs.

**Figure 6:**
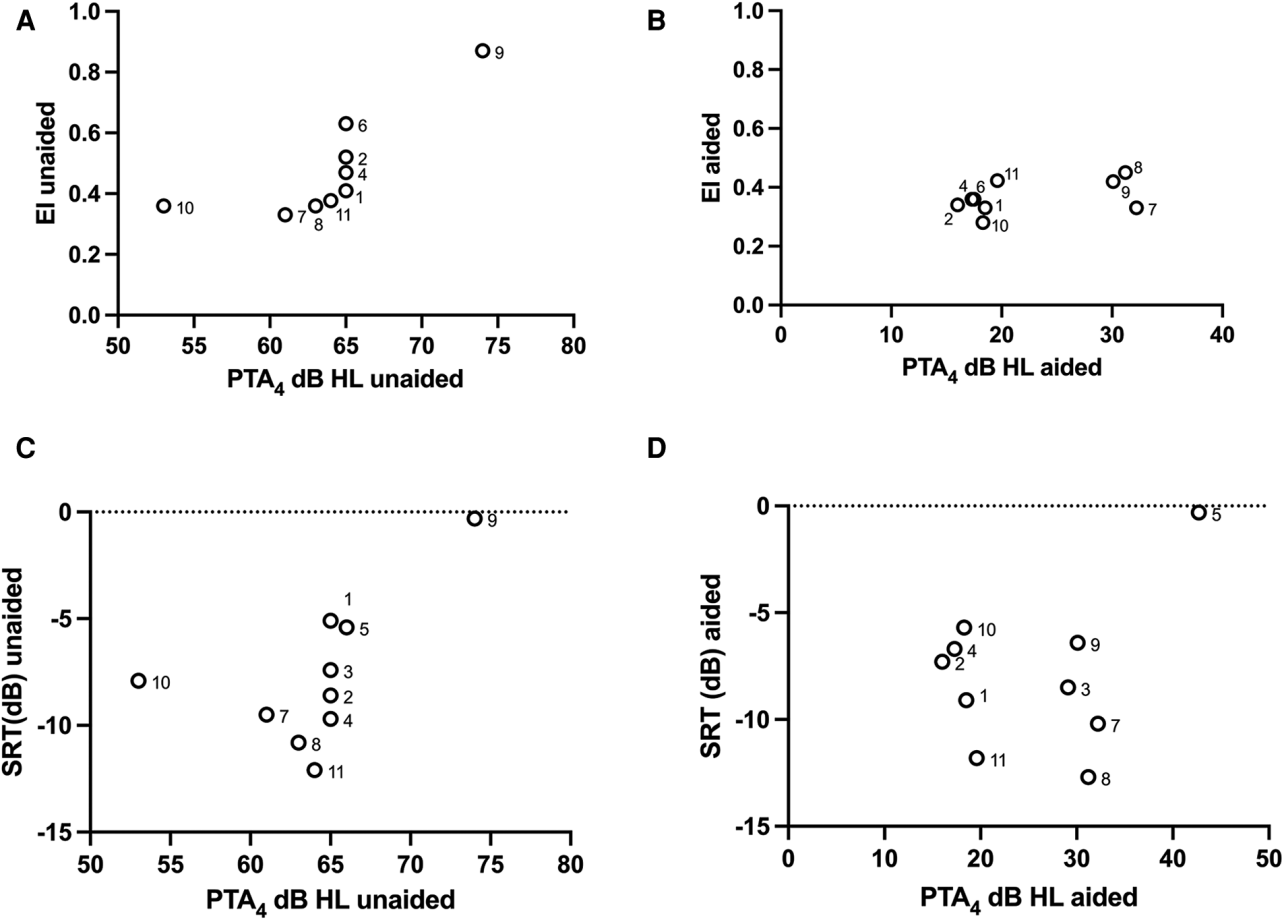
(**A**) Unaided SLA was correlated to unaided PTA_4_ (*p* < 0.05). (**B**) There was no correlation between aided PTA_4_ and aided SLA performance. (**C**) Unaided SRT related to unaided PTA_4_. There was no significant correlation between unaided audibility in the atretic ear and unaided speech recognition thresholds (*p* > 0.05, Spearman correlation). (**D**) No significant correlation was found between aided SRTs and aided PTA_4_ (*p* > 0.05, Spearman correlation).

Age at testing did not have a statistically significant effect on SLA performance on a group level (unaided *ρ* = −0.44, *p* = 0.239; aided *ρ* = 0.29, *p* = 0.45, Spearman correlation) (Figure 5A), or on the benefit from amplification on the task (i.e., the difference between unaided and aided SLA, *ρ* = 0.62, *p* = 0.08, Spearman correlation). The two youngest participants had the worst unaided SLA performance, as well as the most benefit from amplification. Age at first fitting did not correlate with SLA performance in listening condition (aided *ρ* = −0.28, *p* > 0.05; unaided *ρ* = 0.02, *p* > 0.05, Spearman correlation), time of usage per day (unaided *ρ* = 0.05, *p* = 0.92; aided *ρ* = −0.41, *p* > 0.05, Spearman correlation), or duration of device use (aided *ρ* = 0.18, *p* > 0.05; unaided *ρ* = −0.34, *p* > 0.05, Spearman correlation). The participant with the lowest time of usage performed worse in the aided compared to the unaided setting. Unaided SLA was found to be correlated to unaided PTA_4_ of the atretic ear (*ρ* = 0.93, *p* = 0.007, Spearman correlation) (Figure 6A), indicating increased localization accuracy with increasing unaided hearing sensitivity. There was no such correlation for aided hearing thresholds and aided SLA (Figure 6B) (*ρ* = 0.24, *p* = 0.525, Spearman correlation).

### 4.3. SRT in competing speech

One subject did not finish the speech recognition test due to tiredness and was not included in the analysis, i.e., 10 children provided data for this test. Data are presented in Table 3. The mean (SD) SRT was comparable for aided [−7.9 (3.5) dB] and unaided [−7.7 (3.4) dB] listening conditions (*p* > 0.05, Wilcoxon matched pairs) (Table 3 and Figure 4B). SRTs improved with increasing age (unaided *ρ* = −0.88, *p* = 0.002; aided *ρ* = −0.69, *p* = 0.033, Spearman correlation) (Figure 5B) but was not found to be affected by age at first fitting of the BCD (unaided *ρ* = −0.22, *p* = 0.505; aided *ρ* = 0.02, *p* = 0.755, Spearman correlation) or time of usage per day (unaided *ρ* = 0.17, *p* = 0.703; aided *ρ* = 0.12, *p* = 0.793, Spearman correlation). Aided SRTs were found to improve with increased duration of use in years (*ρ* = −0.67, *p* = 0.04, Spearman correlation); however, duration of use in years also correlate with the age of the study participants. Correlation of duration of use was not found in the unaided performance (*ρ* = −0.40, *p* = 0.25). There was a trend toward a correlation between unaided PTA_4_ of the atretic ear to unaided performance (Figure 6C); this was however not significant (*p* = 0.061, Spearman correlation). No correlation was found between aided hearing thresholds and aided SRT (Figure 6D) (*ρ* = −0.10, *p* = 0.785, Spearman correlation).

### 4.4. PEACH questionnaire

Caregivers of all participating subjects filled out the PEACH questionnaire (*n* = 11). The caregiver of one child could not fill in the unaided part of the questionnaire as the study participant used its BCD “during all waking hours.” One subject had not been well during the last week, and one had not been using the BCD during the last week. These questionnaires were excluded from analysis. The total score and the scores for the quiet and noise domains are presented in Table 4. Parents reported significantly higher scores in quiet than in noise for both unaided (*p* < 0.05, Wilcoxon matched pairs) and aided (*p* < 0.05, Wilcoxon matched pairs) conditions, indicating that the study participants might have greater difficulties listening in noisy than in quiet environments. There was no significant difference between unaided and aided scores in any of the domains [Total score *p* = 0.945, quiet domain *p* = 0.375, noise domain *p* = 0.125 (Wilcoxon matched pairs)].

**Table 4:** PEACH scores aided and unaided separated by domain.

Total PEACH score %, mean (SD)		Quiet domain %, mean (SD)		Noise domain %, mean (SD)	
Uniaded	Aided	Unaided	Aided	Unaided	Aided
82.5 (11.3)	81.6 (9.6)	88.5 (10.6)	91.3 (8.0)	70.0 (14.1)	73.5 (11.8)

Results were significantly higher in the quiet domain than in the noise domain in the unaided and in the aided condition (*p* < 0.05, Wilcoxon matched pairs). There was however no significant effect from amplification in any of the domains (*p* > 0.05, Wilcoxon matched pairs).

## 5. Discussion

The aim for this study was to investigate the impact of unilateral bone conduction amplification before 3 years of age on horizontal sound localization and recognition of speech in spatially separate competing speech in children with UAA. Although all study participants improved their hearing thresholds in the aided condition, the intra-individual variability in the benefit for SLA and SRTs was large. Results from this pilot study indicate that fitting with a BCD before the age of 3 does not seem to negatively affect horizontal sound localization accuracy in children with UAA and might be beneficial to some individuals. Results on speech recognition were more diverse, where four individuals showed a worse performance in the aided setting. In a review by Vogt et al. (33), they found that aided hearing thresholds did not approach normal levels in six out of nine included studies and also suggested that an insufficient degree of amplification might be a part explanation for poorer aided speech recognition scores. Several of the study participants in the present study did not reach normal hearing levels of the atretic ear in the aided setting. However, aided hearing thresholds did not significantly affect SRT on a group level.

Age at fitting was not related to SLA or SRT, suggesting that early treatment with a BCD for congenital UAA will not negatively affect these abilities on a group level. Amplification benefits for SLA and SRT were more evident in the younger individuals, who were also fitted at an earlier age (before 1 year of age, subjects 5, 6, and 9). Two of these individuals (6 and 9; no SLA data were collected for subject 5) improved their SLA performance and all three improved their SRTs in the aided condition. Subjects 6 and 9 also had the worst unaided PTA_4_ as well as the worst unaided SLA performance. Agterberg et al. (34) suggested that individuals with worse unaided SLA might benefit more from amplification, consistent with the above stated findings.

Both aided and unaided SRTs increased with increasing age, which might be expected since development of speech recognition is known to continue into adolescence (35). For individuals with congenital UAA, an age effect on SRT is further confirmed by a comparison with data from the current study and adults with UAA [*n* = 12, mean (SD) −10.9 dB (1.4 dB), *p* = 0.008, unpaired *t*-test] (25).

The time of usage of the BCD in this study was quite low and varied between 0.3 to 7.5 h per day but did not seem to influence the results on SLA or SRT. The duration of device use was found to affect aided SRTs but correlates also with the age of the study participants making it hard to draw conclusions from this finding. All study participants had been initially fitted with a BCD on softband before receiving a percutaneous or transcutaneous device, but eight out of 11 subjects had changed to a different system when the study took place. Information on at what age these individuals had received their surgically implanted solutions and audiological data from the previously used BCD on softband with correlated data on time of usage per day was unavailable to the authors. It is possible that children who use their BCD more frequently as toddlers might have greater benefit from the device. Compared to percutaneous devices, passive transcutaneous devices and conventional devices have approximately 10–15 dB lower amplification due to the attenuation of the skin and soft tissues of the scull (36, 37). Even though we found no correlation between aided hearing thresholds and the effect on SLA or SRT from amplification, an effect on the results from the study participants using different BCD systems cannot be ruled out. Due to the small sample size, we were unable to analyze whether the use of different systems might have influenced the results.

Binaural cues, such as interaural time differences (ITDs) and interaural level differences (ILDs) are known to be important for localizing sound in the horizontal plane (38). For individuals with UAA, detection and processing of ITDs and ILDs may be compromised because of reduced audibility in one ear. For horizontal SLA in normal binaural hearing, ITDs have been shown to be dominant, overthrowing ILD and spectral cues for low frequency sounds (39). Monaural spectral cues, resulting from acoustic reflections in the pinna, shoulders, and body are used for localization in the vertical plane in normal hearing listeners (40) and might be of importance for unaided horizontal sound localization in individuals with UAA (8, 11, 33, 34). Here, monaural cues were likely available to the children, but not very prominent given the naturally occurring amplitude modulations of the sound. As such, processing of interaural cues should be important to reach good performance in the task used in the present study. The statistically significant correlation between unaided PTA_4_ and unaided SLA observed needs to be evaluated in a larger sample due to co-variating factors such as the age at first fitting and the duration of time during which amplification had been available. Also, PTA_4_ values of the children tested here were clustered around 65 dB HL (Figure 6A) and the correlation observed depended heavily on two outliers. Notwithstanding that this correlation may not be present in a larger study group, a discussion on the possible influence of the audibility of the atretic ear on localization accuracy is warranted. First, it might be that individuals with less severe hearing loss secondary to UAA might be able to utilize interaural differences for localizing sound in the horizontal plane, since even very low sound levels in the ear with poorer thresholds provide access to interaural localization cues (39). Second, also giving some support to our finding that the PTA_4_ of the atretic ear may be an important predictor of localization accuracy, a similar relationship has been observed in adults with UAA (25) using the same localization technique as in the present study. Third, previous studies in individuals with UAA have demonstrated an increase in localization accuracy at high presentation levels (41, 42). An interpretation of this localization improvement is that both cochleae are stimulated (because of the increased presentation level) and interaural differences may be utilized for sound source localization despite the unilateral hearing loss. This may also be what occurred in the present study in the children with the lowest hearing thresholds.

**Figure 7:**
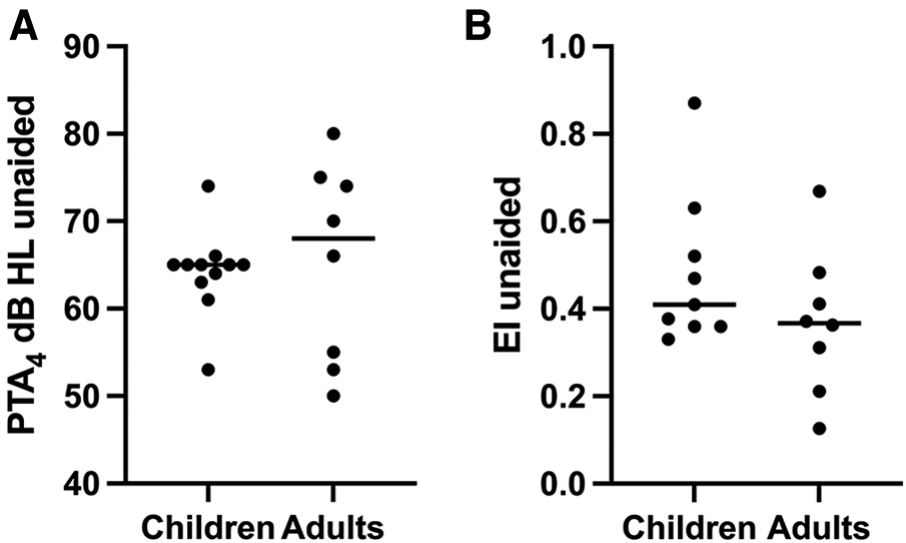
Comparison of unaided PTA_4_ (**A**) and SLA (**B**) between children from the present study and adults from the study by Siegbahn et al. (25). The adults had a tendency toward a higher PTA_4_ and lower EI compared to the children in the present study where the spread tends to approach lower PTA_4_ and a higher EI.

When comparing the localization results of the present study to those of adults with UAA (25), the adults had a tendency toward a lower EI (i.e., better localization) compared to the children in the present study (Figure 7). This is noteworthy, since localization accuracy for normal binaural hearing seems mature at approximately 5–6 years of age (15, 16). The younger participants in the present study showed the worst unaided SLA; however, the effect on amplification was more evident in these individuals as they approached the EI of the older study participants in the aided setting. This could indicate that maturation of SLA is not delayed in these children compared to normal hearing individuals.

Similar to scores from children with moderate unilateral sensorineural hearing loss, results from the overall PEACH scores were lower in the aided and unaided condition than those for normal hearing children in the same age group (22, 31). Parental ratings of aural/oral performance were comparable for unaided and aided listening, suggesting that parents are not able to discriminate whether the BCD is beneficial to the child in the situations described in the questionnaire.

## 6. Study limitations

The statistical power of this study is limited. The authors were only able to present a small sample as only 11 out of 31 individuals that met inclusion criteria decided to take part in the study. Several of the predictor variables co-varied making it difficult to draw conclusions from the results. Aided hearing thresholds also varied in the studied cohort, where five study participants had aided hearing thresholds within the range of mild-to-moderate hearing loss. However, the studied cohort was homogenous regarding hearing thresholds of the non-atretic ear and all study participants had normal BC thresholds in the atretic ear. There was no formal procedure to ensure that the device was fully functioning prior to testing, but since all participants of the study improved their hearing thresholds in the aided setting, we assumed that the BCD was functional. Information on longitudinal device use was not available to the authors.

## 7. Conclusion

Collectively, the results from the current pilot study indicate that the introduction of BCD amplification before 3 years of age in children with UAA does not seem to affect horizontal sound localization accuracy and might result in benefit for horizontal sound localization for some individuals. The effect of early access to amplification on recognition of speech in spatially and symmetrically separated competing speech is more diverse. While there is no significant effect on a group level, some individuals might perform worse in the aided setting. In the future, it would desirable to be able to predict which individuals might benefit more from amplification. The effects of early fitting of a BCD in UAA on spatial hearing needs to be evaluated in a larger sample.

## Data Availability

The raw data supporting the conclusions of this article will be made available by the authors, without undue reservation.
